# Dietary assessment at the confluence of public and planetary health: introduction of the DIEM (Dietary Impacts on Environmental Measures) scoring system

**DOI:** 10.3389/fnut.2025.1678148

**Published:** 2025-10-20

**Authors:** David L. Katz, Marie Lindszewski, Lauren Q. Rhee, Martin C. Heller, Gidon Eshel, Dina L. Aronson, Emily Barrett

**Affiliations:** ^1^Diet ID, Detroit, MI, United States; ^2^Department of Nutrition, Harvard University T.H. Chan School of Public Health, Cambridge, MA, United States; ^3^AgResilience Consulting, LLC, Traverse City, MI, United States; ^4^Bard College, Annandale-on-Hudson, NY, United States

**Keywords:** environmental impact, planetary health, sustainable diets, dietary patterns, diet quality, scoring system

## Abstract

The environmental impacts of foods—including, notably, land use, water use, nitrogen inputs, and greenhouse gas emissions—are substantial and widely varied. Databases that quantify these separate impacts exist, but few aggregate these component measures into a consumer actionable score for the overall environmental impact of given food choices. Whereas data are readily accessible for individual food items, information about overall dietary patterns—combining individual item impacts into a unified, numerical environmental score—is less so. A means of generating an environmental impact score based on real-time dietary intake assessment and/or goal diet selection has not been established. Understanding environmental impacts at this dietary pattern level is especially relevant for informing consumer action. Leveraging available published databases for food environmental impacts and nutrient analysis, combined with novel intellectual property that stratifies dietary patterns into operationally-defined diet types and objectively measured (HEI-2020) diet quality, we developed a unified scale for environmental impacts of overall dietary pattern. We further integrated this approach into real-time dietary intake assessment and personalized goal setting. Here, we introduce the DIEM © (Dietary Impacts on Environmental Measures) scoring system, describe its development, and explore its key implications. The guiding objective is to motivate and empower consumers to reduce their personal dietary environmental footprint while improving diet quality.

## Introduction

Among the important influences on planetary health, human dietary patterns practiced at scale deserve special attention for a number of reasons. First, overall diet quality does not merely influence human health but stands out as the single leading predictor of risk for both all-cause premature death and incident chronic disease (1). Second, dietary patterns that best support human health fortuitously tend to minimize diet related environmental burdens (2).

Third, food production and consumption patterns directly impact most important metrics of planetary health and sustainability, including consumptive water utilization, land use, greenhouse gas emissions, inland freshwater and coastal ocean eutrophication, and soil degradation, among others (3, 4). Of note, food production impacts the environment both directly (through the environmental costs of food production itself) and indirectly (via the upstream environmental costs of fossil fuels and transportation) (5).

Fourth, many environmental costs of food are, from an economic perspective, “externalities,” i.e., costs to which food prices are insensitive. Some extreme examples include beef production in former Amazonian rainforest, or palm oil production in former rainforest in Borneo. The ongoing failure to internalize such “externalities” incentivizes (via product prices) environmental degradation, including of uniquely valuable, iconic ecosystems (6).

Fifth, the net effects listed above, and others omitted, translate into a systematic transformation of biomass on land from its native diversity into a colossal population of cows, pigs, and chickens (7); with a comparable consolidation of biomass now underway for marine species due to commercial fishing and aquaculture (8, 9). And finally, dietary shifts are among the few important impacts on climate and planetary health that are fully actionable by individuals (at least in high-income nations) (10, 11).

Despite such outsized effects on every aspect of planetary health, conjoined implications for personal health, and opportunity for meaningful action by individuals, dietary climate and environmental action has often been neglected in both practice and principle (12, 13)—with the occasional welcome exception (14–16).

Here, we combine a proprietary novel map of dietary patterns (17, 18) incorporating full nutrient-level analysis, with databases of select environmental impacts of foods to devise a scoring method for the overall environmental impact of diverse diets of varying types and qualities. While measures of environmental impact have been applied to dietary patterns before (19, 20) to the best of our knowledge none has been linked directly to real-time dietary intake assessment and personalized goal setting. Below, we elucidate our methods, introduce the DIEM scoring method, illustrate its application with representative scores, and discuss implications at the confluence of human and planetary health.

## Methods

### Use of environmental impact databases

Data were principally sourced from two environmental impact databases: The Harvard T. H. Chan School of Public Health’s food-frequency questionnaire environmental database (FFQED), and Heller et al.’s water scarcity footprint database (WSFD) (21, 22). We selected the FFQED for its “estimates of greenhouse gas emissions (GHGE), use of high-quality cropland (as distinct from rangeland), (and) reactive nitrogen (Nr) (from fertilizer)” for 286 distinct constituent food items, as measured from cradle to farmgate and adjusted to account for edible loss from farmgate to table (23). The FFQED was originally developed to correspond with the 2011 Nurses’ Health Study II food frequency questionnaire to identify correlations between the environmental impacts and long-term health outcomes of four dietary indices: the alternative healthy eating index-2010, an overall plant-based diet index, an unhealthy plant-based diet index, and a healthy plant-based diet index (23). The FFQED has since been published for public use.

The WSFD was selected for its consideration of the environmental impact of irrigation water based on regional water scarcity within the U. S. (and exporting countries for food imports) (22). We added the water scarcity footprint values from the WSFD to the 286 food items in the FFQED, resulting in a singular food environmental impact dataset that includes greenhouse gas emissions, cropland use, reactive nitrogen use, and water scarcity footprints in their corresponding units of measure: kgCO_2eq_, m^2^-yr, g Nr, and liter-equivalents per g of food, respectively.

### Combined (or unified) environmental impact scoring

**Figure 1:**
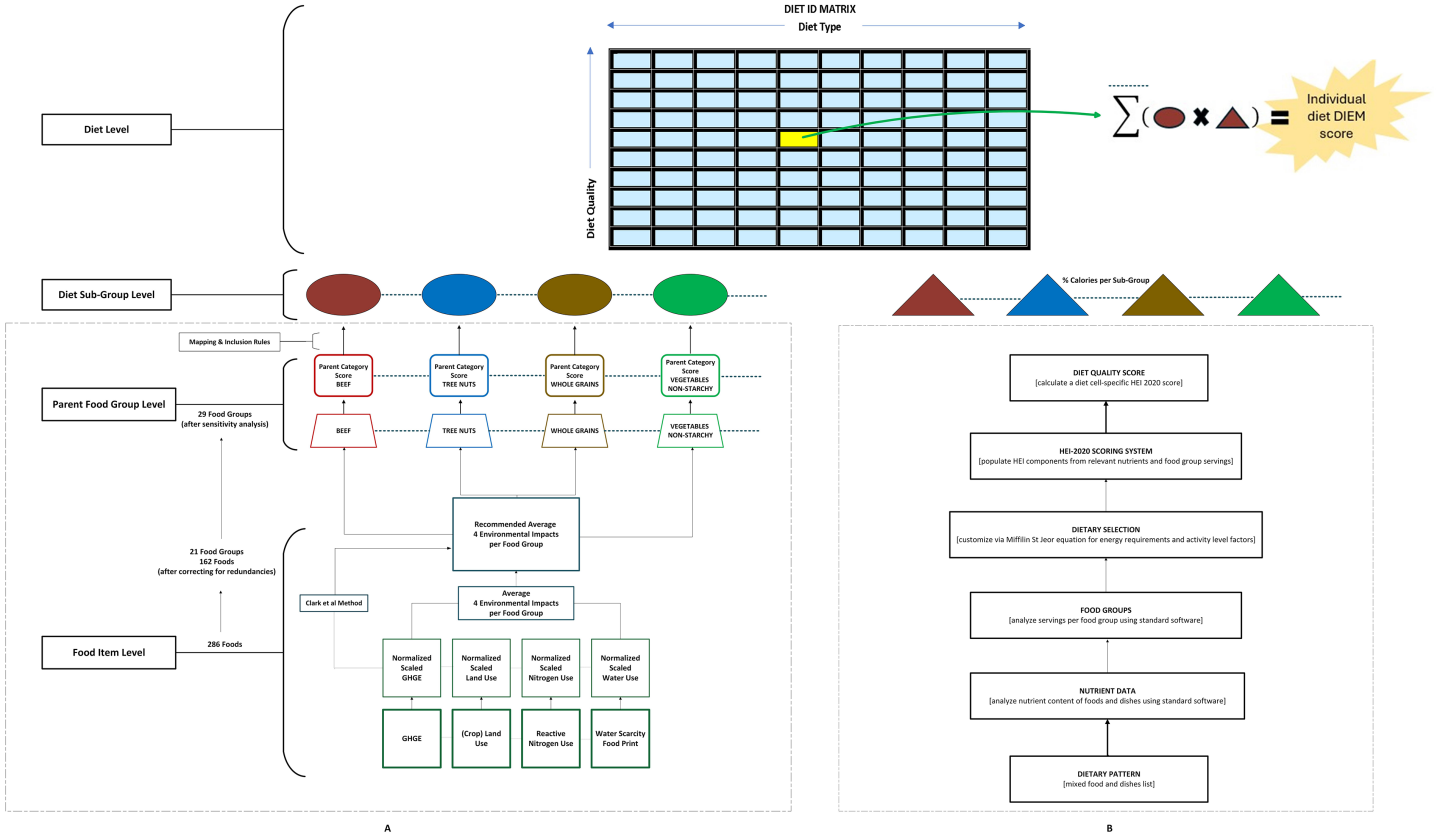
Steps in the generation of DIEM scores. **(A)** The sequence of steps in the generation of environmental impact scores, and **(B)** how they intersect with the corresponding sequence of steps in nutritional analysis and the generation of diet quality scores (i.e., *Healthy Eating Index-2020*) for the same cell in the diet map.

The DIEM scoring method, displayed graphically in Figure 1, illustrates a single, dimensionless measure of environmental impact. To convert the four unit-specific environmental indicators in our list of 286 foods to unitless scores, we utilized Clark et al.’s environmental impact score calculation method (24), as follows: (1) Identify the largest impact score within each environmental indicator; (2) Calculate the scaled impact for each food item and environmental indicator by dividing item specific scores times 100 by the largest impact score; (3) For each food item, average the scaled impacts of all environmental indicators with equal weighting; (4) Determine the “composite environmental impact score” by dividing each food item’s averaged score by the highest averaged score. To assure the appropriateness of equal weighting, we conducted a sensitivity analysis, fixing the weight for greenhouse gas emissions at one, while varying the weights for the other three contributors from 0.5 to 1.0 to 2.0 to account for geographic variation in the primacy of a given factor.

The four environmental impact scores, each with its own unit of measurement, were calculated per unit mass. Food items were categorized according to the 21 food groups outlined by the FFQED. The four rank-ordered environmental scores for each food item were averaged to calculate unitless aggregate scores. We then evaluated whether variations or redundancies in the environmental impact scores within each FFQED food group justified adjustments to the categorization or the exclusion of specific food items. Once the redundancies and variations were corrected (resulting in a total inventory of 162 food items), we took an average of the aggregate environmental impact scores within each FFQED food group. The component measures for environmental impact were weighted equally in generating the composite measure. To confirm ordinal and incremental precision of our data, we examined the consistency between our composite food group environmental scores and those of previously published environmental scoring systems (21, 23, 24).

The resulting 162 food items were mapped to their parent categories corresponding to food groups utilized for nutrient analysis in the Diet ID platform (17). Through a sensitivity analysis, we compared the ranges, medians, and means within each parent category to identify specific foods that needed to be subdivided into separate categories to provide heightened definition. For example, we divided “red meat” into “beef,” “other red meat,” and “pork”; we created new categories for “mollusks” and “crustaceans,” in contrast to the “fish” parent category; “cheeses” and “butter” were individually separated from the “dairy” parent category; we divided “grains” into “whole grains” and “refined grains and grain products”; “corn and olive oil” were separated from “extracted oils—other”; vegetables were divided into “starchy vegetables” and “non-starchy vegetables”; and alcohol was divided into “liquor,” “beer,” and “wine.” After sub-dividing these parent categories, we compared means, medians, and ranges to confirm that inter-category variance exceeded intra-category variance. This confirmation established that parent categories provided the necessary granularity to accurately define food groups that share similar environmental scores while excluding excess detail. After this sensitivity analysis and the addition of “meat alternatives,” we ended up with 29 parent food group categories.

We then averaged the aggregate environmental impact scores of the food items composing each parent category to determine the parent category aggregate environmental impact score (PCS). We again utilized the Clark et al. (24) environmental scoring method to create the raw scores for our parent categories: We identified the highest PCS, divided each PCS by this score, and then multiplied each by 100. This provided an ordinally and incrementally precise method for stratifying all parent categories across the dietary patterns on a 0–100 scale. Per convention, the environmental impact (EI) scores of foods and food categories were ranked on a per-unit-mass basis, while the proportional contribution of foods to overall dietary patterns was weighted on a per-energy basis.

### Application to “diet map”

The Diet ID map (18) includes some 50 dietary patterns to date (25). Entries are derived from the scientific literature and prevailing practice and are characterized by a range from meat-centric (e.g., low-carb; Paleo) to mixed (e.g., flexitarian) to plant-exclusive patterns. Representative foods specific to the dietary pattern are stratified per diet quality tier in deciles (17). They are analyzed using NDSR (Nutrition Data System for Research, software version 2017) for which nutrient and food group data are available at the food level. The generation of nutrient data as described above is then used to calculate an objective, standardized diet quality score, using the Healthy Eating Index (HEI) 2020 (26).

Each DIEM parent category (DPC) is matched to relevant sub-food group data. If there is no exact match to available sub food group data, then qualified foods are determined by inclusion rules. For example, DPC crustaceans are mapped to sub-food group, shellfish. Since shellfish is a broad category that also includes DPC mollusks, an inclusion rule would need to specify shrimp, prawn, lobster, crab, and crayfish. While category matching and inclusion rules allow for a broader application to Diet ID’s dietary patterns, the initial product version may exclude the use of select sub-food groups. See Table 1 for the DPC list.

**Table 1:** DIEM parent categories with illustrative “environmental impact scores,” aggregating scores for land use; site-of-origin adjusted water use; nitrogen inputs (i.e., eutrophication); and greenhouse gas emissions.

Parent category	Parent category score (scaled mean of scaled aggregate EIS per g)	Mean of scaled aggregate EIS per g	Range (low end)	Range (high end)	Standard deviation	# of food items
Beef	100	85.92	73.97	100.00	13.14	3
Other red meat	89	76.64	76.64	76.64	0.00	1
Processed meat	55	46.93	29.47	98.14	35.79	6
Extracted oils—corn and olive	53	45.32	22.25	63.83	25.29	2
Butter	43	37.29	33.82	33.82	0.00	1
Tree nuts	39	33.46	8.44	45.71	16.09	6
Crustaceans	35	30.49	30.80	30.80	0.00	1
Sweets/dessert items	20	17.17	0.95	62.90	24.30	7
Cheese	18.04	15.50	5.14	29.45	9.84	10
Pork	17.97	15.44	14.08	16.72	1.87	2
Poultry	15	12.98	12.82	12.82	1.78	1
Fish	13	11.56	4.43	29.22	8.6	15
Other dairy (except butter, cheese)	10	8.17	2.27	25.16	7.56	12
Alcohol, liquor	8	6.93	6.97	6.97	0.00	1
Extracted oils—other	5.87	5.04	1.49	9.06	2.41	8
Refined grains and grain products	5.53	4.75	2.33	7.71	2.23	6
Alcohol, wine	5.17	4.44	3.65	3.84	0.13	2
Eggs	4.93	4.24	4.18	4.18	0.00	1
Mollusks	4.62	3.97	4.06	4.06	0.00	2
Whole grains	4.02	3.46	1.46	6.27	1.84	6
Fruits	3.56	3.06	0.74	5.97	1.66	20
Legumes	3.01	2.59	0.78	6.01	1.85	8
Beverages	2.74	2.36	0.00	5.44	2.23	14
Vegetables, starchy	2.57	2.21	0.42	5.90	2.14	5
Vegetables, non-starchy	2.39	2.05	0.52	5.53	1.87	21
Meat alternative—tofu	2.06	1.77	1.72	1.72	0.00	1
Peanuts	1.83	1.57	1.53	1.53	0.00	1
Dairy alternatives—all	1.75	1.50	0.26	2.97	1.87	3
Alcohol, beer	1	0.75	0.61	0.90	0.21	2

### Scaling impacts to overall diet

In order to scale the impacts of individual foods to the level of the overall diet, a standard metric is required to determine proportional contributions. The environmental impact databases utilized express impacts per unit mass. Food items and ingredients in the commercial food supply are generally associated with measures of mass, volume, and energy (i.e., calories). For overall dietary pattern, however, neither mass nor volume is routinely available, and the standard measure is energy. Calories are also used in the determination of the HEI score, as a basis for determining nutrient density; these inputs, in turn, derive from the use of calories as the reference standard in both the Dietary Reference Intakes (27) and the Dietary Guidelines (28). Additionally, the foods (e.g., beef, other meats, extracted oils) with the greatest environmental impacts, and thus the largest contributions to DIEM scores, were found in general to be both relatively dense (i.e., mass per unit volume) and energy dense (i.e., calories per unit mass). Empirical testing was done (data not shown) to confirm that the ordinal sequencing of composite, unitless environmental impact measures was consistent whether scaled to energy or mass. Accordingly, to reconcile environmental impact scoring with diet quality scoring within a single platform, environmental impacts were scaled to overall diet by means of their proportional contribution of calories. The selection of energy rather than mass (or volume) was thus predicated on directional and ordinal consistency of scoring with either standard, and alignment with the measure used to define the “size” of a given diet in nutrition. Caloric data effectively function as a weighting coefficient applied to the unit-less DIEM parent category scores (DPCS).

### Testing and refinement of scoring components

Initial testing, using select DIEM parent categories (DPC) and their corresponding median/mean scores and min/max ranges, was applied across the Diet Map, targeting three diet types: Paleo, Standard American, and Vegan. Testing was then expanded to all DPC, with the use of mean scores (DPCS), while meeting a minimum of 95% of a dietary pattern’s total calories (TC-QTDT). Cumulative food group scores per DIEM category (TFGS-DC) were generated to establish a total environmental impact score, i.e., DIEM Score, for each quality tier (1–10, deciles of the Healthy Eating Index 2020) of a given diet type (e.g., Standard American). See Table 2 for calculation variables.

**Table 2:** Variables for DIEM score calculation.

Variable ID	Variable name	Descriptor	Example
QTDT	Quality tier per diet type	Diet quality level of a dietary pattern	Quality Tier 1, Standard American Diet
TC-QTDT	Total calories per quality tier per diet type	Representative 3-day menu, ~2,000 calories each	~6,000 total calories
TFG-FL	Total food group servings per food level	Each representative food has a sum of sub food group values from varied food categories	Cheeseburger (beef, bread, cheese, other vegetable, pickled food) = 8
DPC	DIEM parent category	Food category name	Beef
DPCS	DIEM parent category score	Mean score, ranging between 0 to 100, assigned to each parent category	Beef = 100
TFG-DC	Total food group per DIEM category	Sum of sub food group serving values for a DIEM category	Beef = 2.9 servings
TFGC-DC	Total food group calories per DIEM category	Sum of sub food group calories for a DIEM category	Beef = 234 calories
% TFGC-DC	Percent total food group calories per DIEM category against TC-QTDT	Percent of DPC calories to total diet calories	3.5% beef calories
TFGS-DC	Total food group score per DIEM category	Sum of sub food group scores for a DIEM parent category	Total beef score = 3.4
TDS-QTDT	Total DIEM score per quality tier per diet type	Sum of all TFGS-DC per diet quality level of a dietary pattern	Total DIEM score for quality tier 1, Standard American Diet = 17.2

Specifically, the calculation steps are:

1. Determine the percent of total food group calories per DIEM parent category (TFGC-DPC) relative to total calories per quality tier per diet type (TC-QTDT);
1a. Sum all food calories per quality tier per diet type (TC-QTDT);
1b. Sum all food group calories per DIEM parent category (TFGC-DPC);
1c. Divide TFGC-DPC by TC-QTDT;
1d. Multiply step 1c result by 100 to get percentage;
1e. Repeat step 1 components for each DPC;
2. Determine the total parent category score per quality tier per diet type (TPCS-QTDT);
2a. Divide step 1d percent result by 100;
2b. Multiply step 2a result by DPCS per DPC.
2c. Repeat step 2 components for each DPC.
3. Determine total DIEM Score.
3a. Sum all DPCS.

Steps in the generation of DIEM scores are displayed in Figure 1.

### Statistical analysis

We conducted correlation and regression analyses to examine the variation in DIEM scores in relation to diet type and diet quality. Diet quality, derived from a continuous scale but expressed in deciles, was analyzed with both parametric and non-parametric methods. Specifically, Pearson correlation was used for diet quality as a continuous variable, while Spearman correlation was used for diet quality when treated as an ordinal variable. Diet type was treated as an ordinal variable based on the relative contribution of animal foods to total calories, and non-parametric analysis was applied where appropriate.

Data manipulation and management were conducted using Pandas (29), while NumPy supported numerical operations (30). Statistical analyses, including the Kruskal-Wallis H test and Spearman correlations, were performed with SciPy, and linear regression analysis was executed using scikit-learn (31, 32). For sensitivity analysis of weighting coefficients, n-way analysis of variance (ANOVA) was conducted.

Statistical significance was defined as a two-tailed *p* <0.05, and/or a 95% confidence interval excluding unity.

## Results

While this project was dedicated to product generation rather than hypothesis testing, there were nonetheless associated hypotheses, predicated on the extensive literature characterizing the environmental impacts of foods. The first hypothesis was that DIEM scores would generally increase (i.e., environmental impacts would increase) with the proportion of animal foods, in particular meat, and especially beef, in a given diet (12). The second was that DIEM scores would generally decline as diet quality rose, as higher quality diets rely more on whole plant foods, and less on highly (and ultra) processed foods, the production of which potentially drives up environmental impacts (33). While the direct environmental impacts of food processing are not captured in the DIEM scores (see *Limitations*), the contributions of processed ingredients, such as extracted oils and milled flours, are. In comparing EI scores for parent food categories per unit mass versus energy, ordinal sequencing was generally consistent with the exception of very energy-dense foods typically consumed in small portions (e.g., cooking oils, butter) and very energy-dilute foods typically consumed in relatively large portions (e.g., leafy greens) (data not shown).

**Figure 2:**
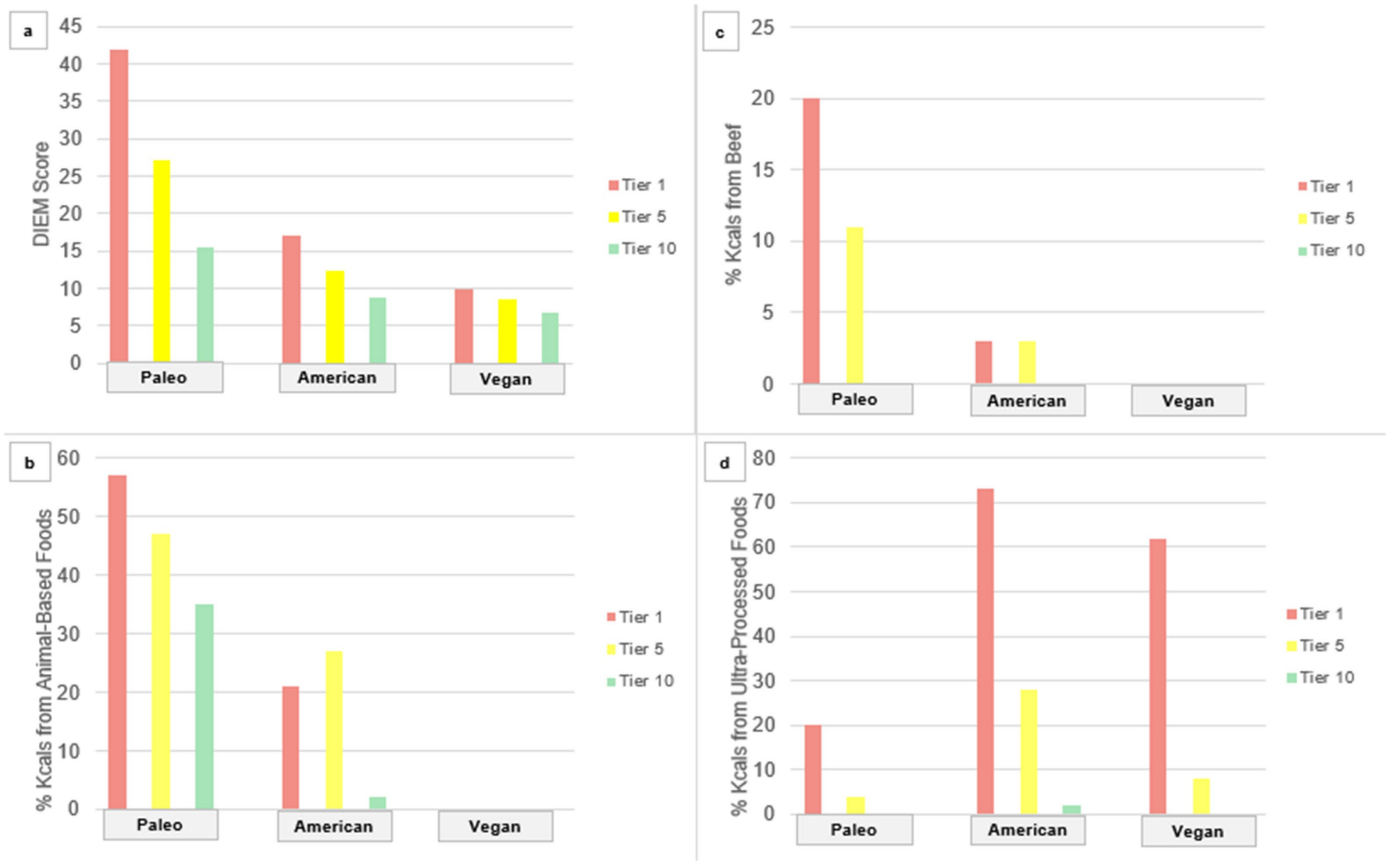
Sample diet summative scores and contributing components across diet quality tiers. **(A)** DIEM Score, **(B)** % Kcals from Animal-Based Foods, **(C)** % Kcals from Beef, **(D)** % Kcals from Ultra-Processed Foods.

Testing the scoring method against the diet map (17, 18) affirmed both hypotheses, as shown in Figure 2. DIEM scores were greatest for a meat-centric Paleolithic dietary pattern; intermediate for a standard American dietary pattern; and least for a vegan (plant exclusive) dietary pattern. In each of the three diets used for testing across the expanse of the diet map, DIEM scores were greatest for lowest quality tiers, and least for highest quality tiers.

The assignment of equal weights to all four components of the aggregate environmental impact score was supported by sensitivity analysis (see Supplementary material). Variation in final DIEM scores was trivial with variation in component weighting. Diet type accounted for approximately 99% of the variance in DIEM scores, whereas alternative weighting coefficients accounted for <1%.

Correlation and multiple regression analysis were conducted to examine the variations in DIEM scores across the full expanse of the diet map. Both diet type and diet quality correlated significantly with DIEM scores, inversely in both cases with diet types numbered from most to least animal-food-centric (see Table 3). Diet type (adjusted Rsq = 0.56) explained more variation in DIEM scores than diet quality (adjusted Rsq = 0.28), in accord with expectation. Together, diet type and quality generated an adjusted Rsq of 0.83. Results of regression analysis are summarized in Table 4.

**Table 3:** Correlation analysis between diet quality, diet type and DIEM score.

Variable	Test statistic	R-squared	*p*-value
Continuous quality and DIEM score (Pearson)	−0.54	0.29	<0.00001
Ordinal quality and DIEM score (Spearman)	−0.62	0.39	<0.00001
Ordinal type and DIEM score (Spearman)	−0.66	0.44	<0.00001

**Table 4:** Regression analysis of diet quality and diet type.

Model	Slope (quality)	Intercept	R-squared	95% CI Lower (quality)	95% CI upper (quality)
Quality only	−1.27	21.36	0.28	−1.54	−0.99
Type only (nominal)	NA	NA	0.56	NA	NA
Quality and type combined	−1.24	20.55	0.83	−1.42	−1.07

A Kruskal-Wallis test was run with DIEM scores as the dependent variable, and both diet type (ordinal) and diet quality (continuous) as the independent variables. The test (H) statistic was 96.74, and the associated *p* < 0.00001.

## Discussion

Measures of environmental impact have been applied to dietary patterns before (19, 20). To the best of our knowledge, however, DIEM is the first scoring system for the environmental impact of food choices to aggregate both horizontally, across component measures (i.e., water use; land use; nitrogen use; greenhouse gas emissions), and vertically from foods to dietary patterns, in conjunction with real-time dietary intake assessment and personalized goal diet selection (17). Combined with novel intellectual property representing diverse dietary patterns in a map of images (18), DIEM represents the first opportunity for an app that can assess current dietary intake and assign environmental impact and diet quality scores simultaneously, and instantaneously. The same methods allow for the assignment of both scores to a range of potential, personalized goal diets, allowing for immediate comparison, and informed selection. The hope, untested at this point, is that such easily accessible comparisons will empower and motivate consumers to choose a goal dietary pattern aligned with their tastes and health goals that imposes the minimal environmental footprint. In the platform as described, any given user is provided a plurality of potential goal diets based on objectively measured diet quality (i.e., the HEI-2020); their personal health objectives and conditions; and personal preference. The DIEM score is then appended to each entry on that list, providing an opportunity to select among dietary patterns already curated for health the option with the smallest environmental footprint.

Of note, we are aware of no other platform that encompasses (a) comprehensive assessment of dietary intake with instantaneous data generation; (b) concurrent identification of options for a personalized goal diet; and (c) concurrent quantification of the environmental impact of each dietary pattern so identified. To the best of our knowledge, this assembly of functions, and the information derived and delivered, are unique to the system described.

Awareness of the linkages among food choices and environmental impacts is limited (34, 35). Changing awareness may not be sufficient to motivate action (36), but is certainly necessary Published literature suggests that changes in knowledge and awareness of the environmental impacts of foods and diets can alter intentions for dietary behavior change (37, 38).

From the world of business comes the observation that “we manage what we measure” (39). This pertains at least comparably in the domain of medicine and public health, where important parameters are measured routinely and treated “to goal,” the constellation of “vital signs” in the vanguard of these. We postulate that this expression pertains as well to the environmental impacts of personal actions, i.e., if measured routinely, our habitual environmental footprint would demand attention and invite action.

The Latin phrase “*carpe diem*,” translating to “seize the day,” pertains well to the imperatives of planetary health as, with each passing day, the opportunity to save what remains of nature, fragile ecosystems, biodiversity, and the hospitability of familiar climate patterns all diminish. The name “DIEM” was informed by such considerations.

The DIEM metric generates a clear, summative score for the environmental impacts of overall dietary pattern. The metric performs as expected, improving with shifts from animal foods to plants, and from low to high quality diets. In application, DIEM scores are unit free on a relative 10-point scale for ease of interpretation and use, but are derived from robust, peer-reviewed scales established for the included component measures.

The DIEM metric is intended for public use. It is also intended for use with the novel intellectual property established to advance diet quality photo navigation (18, 19). In this context, the score can be generated in as little as 1 min as part of dietary intake assessment; and DIEM scores may be attached to dietary recommendations responsive to personal health goals, ethnicity, and preferences. Routine inclusion of environmental impact scores as part of comprehensive dietary intake assessment and guidance is hoped to serve as a cue to action, and a basis for blending the imperatives of personal and planetary health in selecting a diet goal.

### Limitations

There are, of course, limitations to the DIEM metric. There is an environmental impact of food packaging as well as food itself, and the metric does not account for these impacts. Some impacts—such as the dispersal of plastics in the environment from single-use plastics- are fully independent of the four impact domains captured in the DIEM metric. Attention to such additional impacts by the environmentally conscious consumer is obviously warranted. Additionally, packaging (along with processing, transporting, etc.) may contribute to the four impacts that are captured, and the DIEM metric does not incorporate these additional “post farm gate” impacts. Such impacts typically account for no more than 10–15% of total greenhouse gas emissions, with modest variability among food categories (5). Accordingly, whereas the absolute environmental burdens would differ somewhat from the final all-inclusive burdens described here were post farm gate impacts incorporated, the relative standing of all considered items, including compound ones, would not vary to any appreciable degree.

Similarly, the metric accounts for regional differences in water demand to give national average water scarcity footprint scores for U. S. foods, but it does not directly account for distance traveled by a food from field (or factory) to table. This is a complex topic in its own right, with some clear advantages to local sourcing in many cases, but not all (40).

Most importantly, the DIEM scores do not directly account for the effects of food processing. Processing impacts are represented only to the extent that “derived” foods and ingredients, such as extracted oils and milled flours, are represented as distinct entries from their parent foods (e.g., seeds and grains, respectively) in the accessible databases of both environmental impacts and nutrient analysis.

Of note, DIEM scores vary much more with diet type than quality (see Tables 3, 4), and the impacts of processing such as extraction, extrusion, distillation, mixing, packaging, etc.- introduce an error factor apt to pertain comparably across all diet types, but not all tiers of diet quality. Because across diet types this “error” factor is fixed and thus introduces no bias, it likely changes absolute values of DIEM scores, but not their relative magnitudes, which is the only meaningful measure for this dimensionless score.

However, blindness to the environmental impacts of processing is apt to affect the relationship between diet quality and DIEM scores more, because there is, in general, much more processing of food products in the lower quality tiers than the higher (data not shown). Diet quality correlated as expected with DIEM scores, but this correlation is likely biased toward the null by the inability to capture fully the effects of processing (and packaging). Accordingly, we posit that with better accounting for the environmental impacts of food processing, the correlation between diet quality and DIEM scores would strengthen; the correlation between diet type and DIEM scores would remain largely unchanged; and the total variation in DIEM scores explained by the combination of diet type and quality would increase. The ordinal distribution of scores, however, would not be materially affected.

Finally, the DIEM score assumes an equal weighting across the four environmental metrics combined in the scoring system. In other words, the environmental metrics are assumed to have equal importance; such ‘ranking’ of environmental impact categories is inherently subjective and will vary by population. For example, in regions with extreme water scarcity, water use impacts may carry greater local importance than climate change. The intention of the DIEM metric is to guide selection of diets generally, or universally: equal weighting is perhaps the most transparent way of combining metrics.

## Conclusion

The DIEM scoring system represents a novel advance in the understanding and management of food-related environmental impacts, combining real-time dietary intake assessment, personalized dietary goal setting, and diet quality scoring with environmental impact scoring. By creating a comprehensive, accessible method for quantifying the environmental cost of dietary patterns—one that aggregates across multiple metrics and scales up from individual foods to entire diets—while inviting real-time comparison and selection, this effort is directed at bridging the gap between awareness and action. The expression that “we manage what we measure” underpins this work, and the hope is that DIEM will serve as a catalyst for change, empowering individuals and institutions alike to align food choices with both personal health goals and planetary sustainability. The system described allows for the superimposition of environmental impact scores on a plurality of personalized diet pattern recommendations for personal health. Studies employing this system will be able to demonstrate if and how quantitative variation in the environmental footprint of dietary options changes behaviors in the short term and long.

While limitations in accounting for factors like packaging and transport remain, these do not undermine the core utility of the DIEM score in offering a comparative view of dietary impacts. In an era where the clock on climate change is ticking ever louder, the ability to make informed, impactful choices about what we eat could provide one of the more accessible avenues for meaningful climate action. The DIEM system invites users to seize the day, and take immediate steps to reduce their dietary footprint—one meal at a time.

## Data Availability

The datasets presented in this article are not readily available because data generated during the current project are available upon reasonable request. Requests to access the datasets should be directed to Lauren Rhee, Lrhee@dietid.com.
